# Cell cycle progression in response to oxygen levels

**DOI:** 10.1007/s00018-014-1645-9

**Published:** 2014-05-25

**Authors:** Brian Ortmann, Jimena Druker, Sonia Rocha

**Affiliations:** Centre for Gene Regulation and Expression, College of Life Sciences, University of Dundee, Dow Street, Dundee, DD1 5EH UK

**Keywords:** Hypoxia, CDKs, Proly-hydroxylases, HIF, JMJC

## Abstract

Hypoxia‚ or decreases in oxygen availability‚ results in the activation of a number of different responses at both the whole organism and the cellular level. These responses include drastic changes in gene expression, which allow the organism (or cell) to cope efficiently with the stresses associated with the hypoxic insult. A major breakthrough in the understanding of the cellular response to hypoxia was the discovery of a hypoxia sensitive family of transcription factors known as the hypoxia inducible factors (HIFs). The hypoxia response mounted by the HIFs promotes cell survival and energy conservation. As such, this response has to deal with important cellular process such as cell division. In this review, the integration of oxygen sensing with the cell cycle will be discussed. HIFs, as well as other components of the hypoxia pathway, can influence cell cycle progression. The role of HIF and the cell molecular oxygen sensors in the control of the cell cycle will be reviewed.

## The HIF system

Hypoxia‚ or decreases in oxygen concentration‚ results in the activation of a number of different responses both at the cellular and whole organism level [1]. These responses include changes in gene expression, which allow the organism to manage efficiently the hypoxic stress. A major advance in the molecular understanding of the hypoxic response was the discovery of the hypoxia inducible transcription factor (HIF). HIF is a heterodimeric transcription factor, which consists of a constitutively expressed HIF1β (gene name ARNT for Aryl hydrocarbon receptor nuclear translocator) and an oxygen sensitive HIFα subunit [2].

**Fig. 1:**
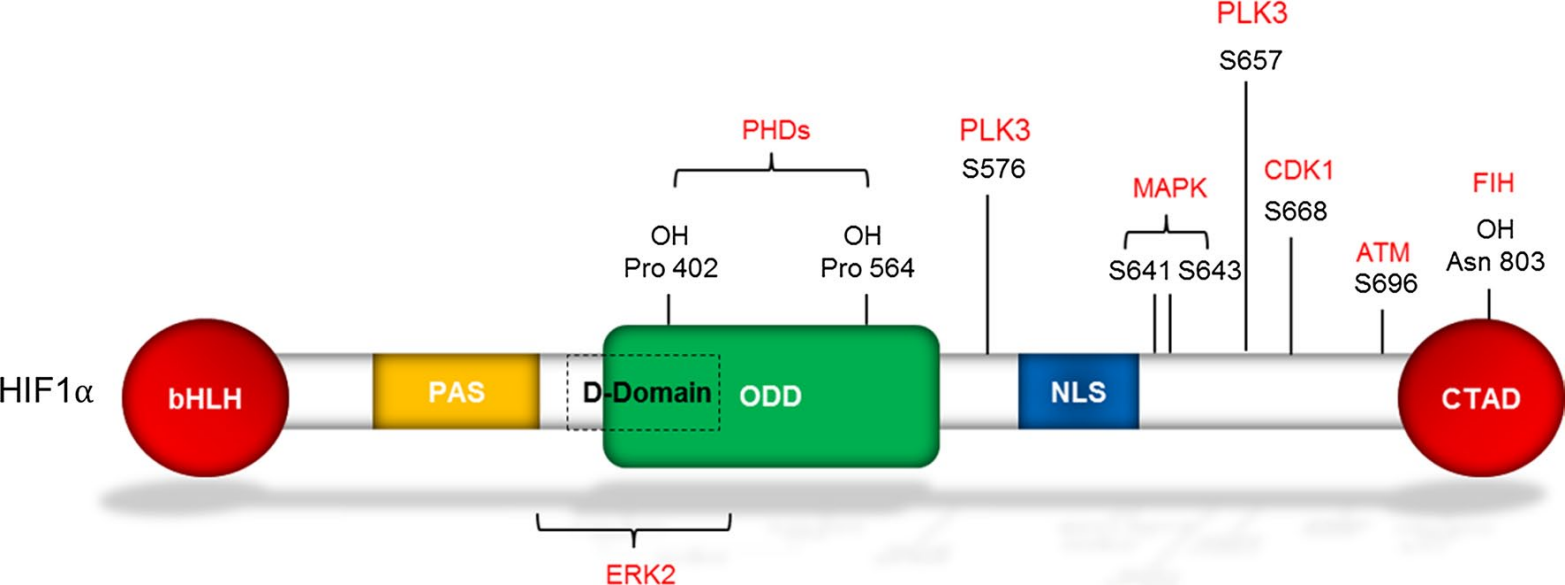
Structure of HIF1α. Diagram highlighting the structural domains of HIF1α. Postranslational modifications including hydroxylation and phosphorylation including the proteins which mediate this function are shown in *red*. The sites which are shown to be phosphorylated are most relevant with cell cycle regulation [91, 161–163]. Also shown on the structure is a putative D-domain which serves as a docking site for ERK2 [164]

Currently, there are three known isoforms of the HIFα subunit (HIF1, 2 and 3). All of the α subunits contain similar domains (Fig. 1), most notably the presence of a basic helic-loop-helix (bHLH)-Per-Arnt-Sim (PAS) domain which is essential for its interaction with HIF1β [3]. In addition, all three isoforms contain an oxygen-dependent degradation domain (ODD), which sensitizes these proteins to destruction in the presence of oxygen [4]. HIF1α and HIF2α both contain C-terminal transactivation domains, whereas HIF3α does not. Currently, studies investigating HIF3α, suggest that it acts as a dominant negative inhibitor of both HIF1α and HIF2α [5]. Newly published data from 2014 from a study in zebrafish have shown that HIF3α does in fact have transcriptional activity [6]. Due to the high sequence similarity between HIF1α and HIF2α, they have been shown to share several target genes; however, they also have their own set of specific targets allowing HIFα isoforms to have differential functions [7].

**Fig. 2:**
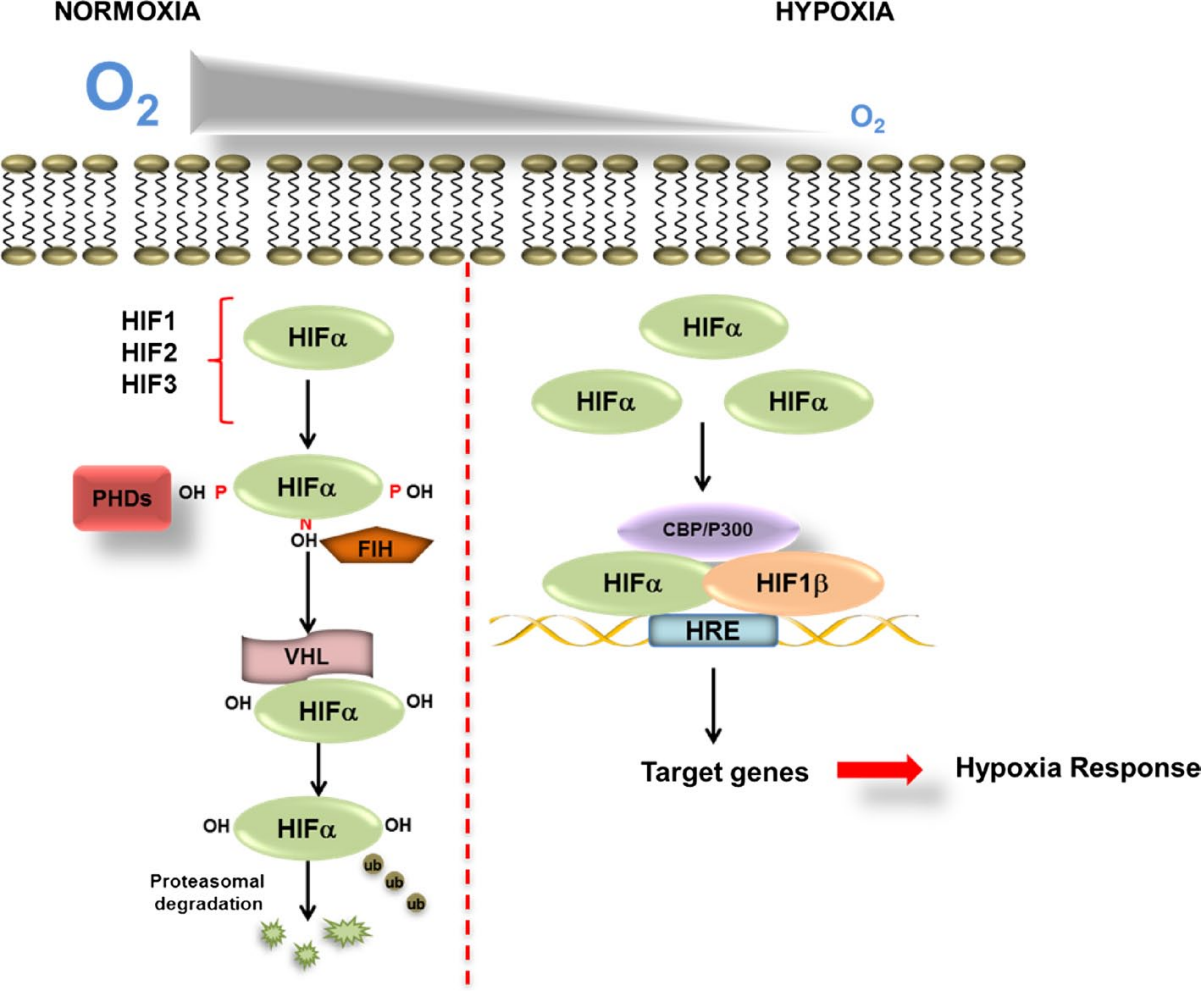
Regulation of HIF in normoxia and hypoxia. Diagram showing the key components in the regulation of HIFα during normoxia and hypoxia. During normoxia, PHD enzymes and FIH use molecular oxygen as well as cofactors to hydroxylate HIFα on proline and an asparagine residue, respectively. Hydroxylation of proline residues within the ODD domain of HIFα mediates the binding of the VHL E3 ligase which polyubiquinates HIFα and hence targets it for proteasomal degradation. In hypoxia, when oxygen levels are decreased PHDs and FIH are inhibited leading to HIFα stabilization and dimerization with its transcriptional partner HIF1β. HIF can then activate transcription of target genes and recruit of co-activators p300/CBP

The expression of HIFα subunits is regulated primarily at the posttranscriptional level through hydroxylation-dependent proteasomal degradation (Fig. 2). More recent studies have suggested that HIF1α is also subject to lysosomal-mediated degradation [8]. Lysosomal-mediated degradation is thought to be independent of hydroxylation. In addition, HIF1α is also regulated at the level of transcription and translation by NF-κB and the mammalian target of rapamycin (mTOR), respectively [9–12]. Furthermore, the stability of HIF1α mRNA has been shown to be dependent on P-body function [13].

In normoxia, when cells are well oxygenated, HIFα is subject to hydroxylation within its ODD domain by a family of dioxygenase enzymes called prolyl hydroxylases (PHDs) [14]. HIF1α is hydroxylated at proline 402 and 564, whereas HIF2α is hydroxylated at proline 405 and 531 [15, 16]. PHD1, 2 and 3 have been shown to regulate the HIFα [16]. Biochemical analysis of the PHDs has shown that PHD2 has the highest affinity for HIF and is currently thought to be the master regulator of the hypoxia response [17]. PHD1 and 3, on the other hand, have been found to have a preference for HIF2α [16]. PHD activity also requires α-ketoglutarate (a-KG) and Fe^2+^ as molecular cofactors and molecular oxygen as a co-substrate [18, 19]. The sensitivity to oxygen makes PHDs the perfect sensors for cellular oxygen levels. In normoxia, hydroxylation of HIFα by PHDs creates a binding site for the von Hippel–Lindau (VHL) tumour suppressor E3 ligase complex [20]. Interaction with VHL leads to Lys48-linked polyubiquitination and degradation via the proteasome [21]. In addition to VHL, it has also been shown that receptor for activated protein kinase C (RACK1), a protein which plays a role in diverse molecular processes such as signal transduction [22] could directly compete with heat shock protein 90 (HSP90) for binding of HIF1α. Binding of RACK1 to HIF1α leads to Elongin C dependent degradation of HIF1α [23].

In hypoxia, when cells are exposed to reduced levels of oxygen, PHD activity is reduced leading to HIFα stabilization and dimerization with its transcriptional partner HIF1β. Dimerization leads to translocation to the nucleus and binding to consensus hypoxia responsive elements (HRE) within the promoters or enhancers of HIF target genes [24, 25].

In addition to regulation of HIFα turnover by the PHDs, fine-tuning of HIF-dependent transcription is achieved through an asparagine hydroxylation within the C-terminal transactivation domain (CTAD) of HIF1α and HIF2α, mediated by a ubiquitously expressed Factor Inhibiting HIF (FIH) protein [26]. FIH hydroxylates HIF1α at asparagine 803 and HIF2α at asparagine 847. Asparagine hydroxylation within the CTAD prevents HIF interaction with its transcriptional co-activators p300 and/CREB–cAMP-response element binding protein (CBP) and as a consequence prevents full target gene activation [27]. In a similar manner to the PHDs, FIH requires α-KG and Fe^2+^ as cofactors, and molecular oxygen as a substrate to carry out this hydroxylation. However, in contrast to the PHDs, FIH is much less sensitive to oxygen levels and some studies have suggested that FIH acts as more of a redox sensor within the cell, as it is strongly inhibited by peroxide [28].

HIF regulates the transcription of a myriad of target genes that are involved in metabolism, autophagy, apoptosis, angiogenesis, and cell proliferation [29, 30]. Ultimately, the transcriptional program initiated during hypoxia aims to promote cell survival, and to turn off highly energy consuming processes such as translation and cell proliferation [31, 32]. In many types of cancer as well as in other disease states such as heart failure and kidney disease, there is aberrant HIF activity [33, 34]. Solid tumours are extremely hypoxic and cells in these regions have been found to be resistant to chemo and radiotherapy [35].

## The cell cycle

One of the most important and energy consuming processes within the cell is the cell cycle, which ultimately results in cell division and the inheritance of genetic information into the daughter cell. The cell cycle is divided into four different phases and begins with Gap 1 (G1) phase. G1 phase together with synthesis (S) and Gap 2 (G2) phase, also known as interphase, involves cell growth, biosynthesis of mRNA, protein and, organelles that are all needed for DNA replication [36]. Once a cell enters S phase, it is committed to cell division. Following interphase, cells enter mitosis or M phase, which is the process by which chromosomes are separated into two daughter cells, a process that is dependent on the formation of a mitotic spindle [37].

The cell cycle is a critical process to the cell, and must be strictly regulated to avoid hyperproliferation and genetic instability that can lead to disease states such as cancer [38]. The vast majorities of cells in an adult organism exist in a quiescent state and therefore do not express many cell cycle-associated genes. In quiescent cells, transcription and translation of factors needed to initiate the cell cycle are repressed in a cell-specific manner by the retinoblastoma protein (pRB) [39]. For cells to progress through the cell cycle, pRB must be inactivated. Loss of function of RB leads to deregulated cell proliferation and promotes tumour progression [40]. Inactivation of pRB is achieved through the activation of cyclin-dependent kinases (CDKs, see below). In its hypophosphorylated state, RB is found tightly bound to E2F family of transcription factors, thus preventing their transcriptional activity [41]. For an in-depth review of E2F and RB, please see [42].

CDKs are a family of small molecular weight serine threonine kinases [43]. Activation of CDKs is highly dependent on their association with a regulatory cyclin, which in turn is produced in response to a mitogenic signal [44]. In addition to cyclin binding, CDKs are regulated by phosphorylation. To achieve full activation of the kinase activity, CDKs must be phosphorylated on a threonine residue close to the active site by a cyclin-dependent kinase activating kinase (CAK) [45]. CDK activity is also regulated by inhibitory phosphorylation by Wee1 and by phosphatase activity of the Cdc25 family of phosphatases [46, 47].

Regulation of CDK activity, and hence cell cycle progression, is not only achieved through post-translational modification. Proteins such as p21 (cyclin-dependent kinase inhibitor 1A) and p27 (cyclin-dependent kinase inhibitor 2A) can also inhibit CDK function [48, 49]. These proteins work by directly binding to CDKs and inhibiting the interaction of its activating cyclin. Binding of p21 to CDK results in growth arrest and, in addition, plays a role in cellular senescence [50]. The expression of p21 is tightly controlled at the transcriptional level through p53 and also by Myc-dependent repression [51]. Activation of p53 through a variety of different stimuli such as ionizing radiation results in p53-mediated G1 cell cycle arrest. In addition, p21 is also tightly regulated, at the protein level, by ubiquitin-dependent and independent degradation pathways [52].

When cells are stimulated by growth factors that act as mitogenic signals, this induces cell cycle entry and CDK activation. It is thought that cyclin D/CDK4 complexes are responsible for entry into G1 [53]. In response to a growth signal, and activation of pathways such as Wnt and Sonic hedgehog (Shh), this results in the induction of the Myc transcription factor [54]. Activation of Myc is known to drive proliferation, and Myc levels and activity have been found to be abnormal in many types of cancer [55]. Myc plays a role in a wide variety of cellular processes such as cell growth, apoptosis and differentiation, but one of the first recognized functions of Myc is in the regulation of cell proliferation [56]. Myc can transcriptionally downregulate cell cycle inhibitors such as p21, and also increase the expression of positive regulators namely the cyclin D, by mechanisms including transcription and translation [57, 58]. Increased expression of cyclins leads to an increase CDK activity, phosphorylation of RB, and release of E2F transcription factors that promote cell cycle entry [59].

## HIF-dependent control of the cell cycle

For cells to proliferate, they must enter the cell cycle, a process known to be highly energy demanding and very tightly regulated. Oxygen is a fundamental ‘nutrient’ in the process of oxidative phosphorylation. Oxidative phosphorylation is the cell’s greatest net energy producing process, when compared with glycolysis. As such, for a cell to commit to cell division, it must overcome energy checkpoints [60]. Therefore, it would make sense that components of the oxygen-sensing system could directly influence cell cycle progression.

Many different cell types respond to hypoxia by inducing cell cycle arrest [61–63]. Increased cell proliferation during hypoxia would increase the O_2_ consumption within the population, leading to an even more hypoxic environment. Early studies on the effect of hypoxia on the cell cycle revealed the induction of a reversible cell cycle arrest when cells were exposed to prolonged hypoxia [41]. Flow cytometry analysis showed that there was a decrease in the percentage of cells in S phase and an increase in the percentage of cells in G1 phase. Entry in S phase is dependent on CDK activity towards pRB and it was observed that hypoxia caused a decrease in CDK activity and consequently, an accumulation of pRB in its hypophosphorylated growth suppressive forms [41].

 Although pRB is controlled by phosphorylation by the CDKs, it has also been shown that its activity is specifically regulated by the phosphatase activity of PP1 [64]. Earlier studies demonstrated that during late M phase, pRB is converted to its growth suppressive hypophosphorylated form through the action of PP1. PP1 activity is increased in hypoxia, working thus as an additional block to cell cycle entry [41].

The decrease in CDK activity in hypoxia is accompanied by an increase in the levels of the CDK inhibitor p27 [65]. The regulation of p27 during hypoxia is still an area of debate within the hypoxia field. Several groups have shown that the induction of the cell cycle inhibitors p21 and p27 is dependent on HIF1α [62, 66]. Work using mouse embryonic fibroblasts and splenic B lymphocytes demonstrated that cell cycle arrest during hypoxia was HIF1α dependent but p53 independent. In addition, loss of HIF1α leads to progression of cells into S phase and a loss of the induction of p21 and p27 [65]. In this way, it is proposed that the expression of p27 is dependent on HIF1α. Conversely, additional studies have shown that p27 is regulated independent of HIF1α, through transcriptional regulation of its proximal promoter [67]. Although the regulation of p27 during hypoxia is still an area of debate, it is clear that p27 plays a critical role in the cell cycle arrest observed during hypoxia. It may be that regulation of p27 expression occurs in a cell type-specific manner, where in some cell types, it is regulated directly by HIF1α, and in others, it is independent of this transcription factor.

**Fig. 3:**
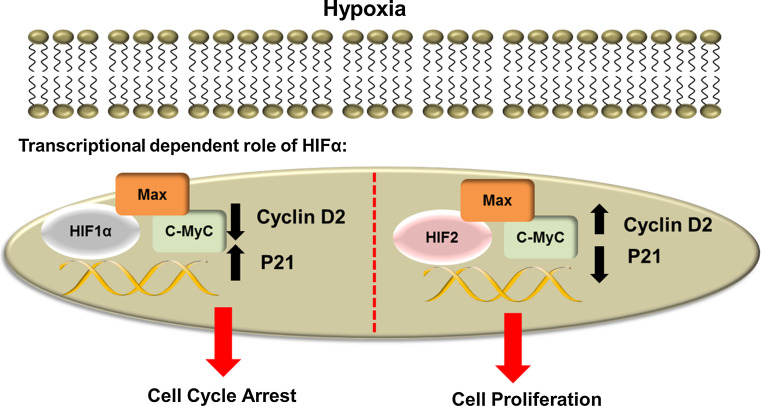
Transcriptional-dependent role of HIFα in the cell cycle. When HIF1α is induced, HIF1α can disrupt complex formation between c-Myc and Max and hence affect c-Myc transcription. Disruption of c-Myc complexes leads to a decrease in c-Myc transcription and repression leading to the induction of p21 expression which in turn causes cell cycle arrest. HIF2α on the other hand enhances the interaction between c-Myc and Max and as a consequence increase the transcriptional activity of c-Myc. As a result of this, there is an increase in cyclin D2 expression and a decrease in p21 leading to increased cell proliferation

Hypoxia can also result in the induction of p21 in many cell types leading to a G1 arrest [66, 68]. The transcriptional regulation of p21 by hypoxia involves a complex interplay between HIFs and also c-Myc (Fig. 3). In normal cells, c-Myc is induced in response to a growth signal and acts as a transcriptional activator or repressor depending on the context. It has been shown to promote transcription of cyclin D2 through its interaction with Max and E box domains [56, 69]. On the other hand, c-Myc has been shown to repress transcription of other cell cycle-related genes such as p21 and p27 [57, 70]. HIF1α induction during hypoxia can lead to a disruption between c-Myc and Max. As a consequence of this, there is less promoter binding of c-Myc, resulting in a decrease of c-Myc dependent genes such as cyclin D2. Loss of c-Myc binding to DNA also has the effect of relieving repression of genes such as p21 and p27, allowing them to inhibit cell cycle progression [62]. As well as regulating the cell cycle during hypoxia, it has also been shown that HIF1α can regulate cell cycle progression during normoxia. Work from our lab has shown that in the absence of HIF1α, Sp1, a transcription factor that plays a role in the regulation of differentiation, cell growth and apoptosis can compensate for the loss of HIF1α and induce p21 mRNA expression [68].

HIF2α has been shown to have an opposing effect to HIF1α on c-Myc activity (Fig. 3). Induction of HIF2α results in increased stabilization of c-Myc-Max complexes, and hence an increase in DNA binding. As a consequence of this, c-Myc dependent transcription of targets such as cyclin D2 and E2F1 increase, and p21 and p27 are repressed. It is thought that this is one of the mechanisms by which HIF2α promotes hypoxic cell proliferation and tumourigenesis [71]. In addition to enhancing c-Myc transcription, HIF2α has also been shown to regulate Oct4 [72], a gene that can promote stem cell phenotypes and induce cyclin D1, hence promoting cell growth. More recently, HIF2α was shown to engage mTORC1 activity, and promote cell proliferation via the regulation of the amino acid transporter, SLC7A5 [73]. These findings suggest that HIF2α may play a critical role in promoting cell proliferation in hypoxia.

As well as regulating the transcription of a multitude of different protein targets, the HIFs have also been shown to regulate the transcription of several microRNAs (miRNAs) [74, 75]. MicroRNAs are short noncoding RNAs, normally around 22 nucleotides in length, which have the ability to regulate multiple mRNAs and are often found to be misregulated in cancer [76]. Of particular interest to the control of the cell cycle is miR210. miR210 is upregulated in a transcriptional-dependent manner by HIF1α, in vitro and also in vivo. mir210 is often used as a marker of tumour hypoxia and patient prognosis, particularly in head and neck cancers [77, 78]. In addition to miR210’s roles in the response to hypoxia, miR210 also been shown to regulate cell cycle progression. In a study by Zhang et al., it was shown that miR210 regulates E2F3, a transcription factor critical for cycle progression [79]. When miR210 is induced, E2F3 is strongly downregulated at the protein level. As a consequence, cell cycle progression is inhibited [79].

On the other hand, miR210 can also promote cell cycle progression. The mechanism through which miR210 can promote cell cycle progression is mediated via downregulation of a Myc antagonist protein called Mnt [80]. Mnt mRNA was found to contain multiple binding sites for miR210 in its 3′UTR. Downregulation of Mnt increased Myc transcriptional activity and hence cell cycle progression. Overall, these studies showed that, as well as modulating proteins that control cell cycle, HIFs can also change the miRNA signature of the cells, which in turn can influence cell cycle progression.

**Fig. 4:**
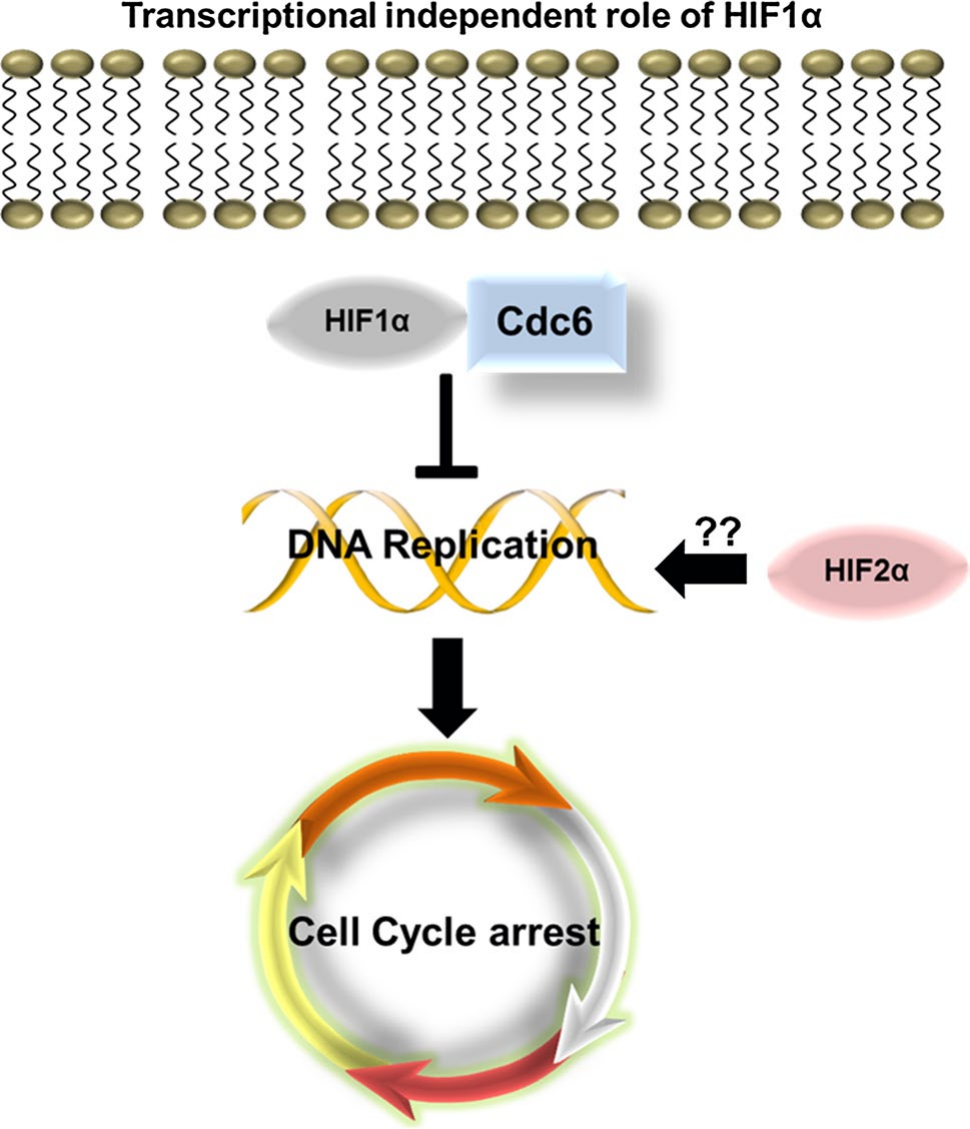
Transcriptional-independent role of HIF1α in cell cycle regulation. When HIF is induced during hypoxia HIF1α can interact with Cdc6 a key component of the DNA replication machinery. Interaction of HIF1α with Cdc6 prevents activation of the MCM helicase and hence inhibits DNA replication. The role of HIF2α in this process has never been investigated

In addition to the transcriptional roles that HIF plays in regulating the cell cycle, recent studies have begun to unravel transcriptional-independent roles of HIF1α in regulating cell cycle progression (Fig. 4). Hypoxia has been shown to block DNA replication [81]. DNA replication must be tightly controlled to avoid errors that could be catastrophic for cell viability, but can also lead to disease states such as cancer. Early studies on the effects of hypoxia using murine tumour cells demonstrated that low oxygen can result in DNA over-replication and the cells became more metastatic [81]. Further to this, additional studies showed that hypoxia caused the activation of ATR and its downstream target checkpoint kinase 1 (Chk1) [82]. Although the mechanism of ATR activation in hypoxia is not fully understood, a recent study showed that ATR-interacting protein (ATRIP), a protein crucial for the activation of ATR, was a direct target of HIF1α. Loss of ATRIP by RNA interference of HIF1α or ATRIP abolished the activation of ATR and Chk1 in hypoxia indicating this could be the mechanism of hypoxia-induced ATR activation [83]. Activation of ATR results in G1-S cell cycle arrest [84]. In addition, other DNA damage response proteins are also activated in hypoxia, including Rad17, histone variant 2A, and H2AX [85].

In a recent series of studies, Semenza and colleagues have shown that the minichromosome maintenance (MCM) proteins are negative regulators of HIF1α [86]. MCM proteins are key components of the DNA helicase, which mediate DNA replication, an essential process prior to cell division. The process of DNA replication is tightly controlled and involves several steps of origin recognition, licensing and finally activation [87]. This process begins in G1, with the formation of a pre replication complex, which determines all the areas of potential origin firing. The pre replication complex consists of six subunits (origin of recognition complex, Orc1-6). Binding of the pre replication complex results in the recruitment of Cdc6, which subsequently attracts the MCM helicase, consisting of MCM2–7. The MCM helicase unravels the DNA, however, replication is not initiated until S phase, when Cdc6 is phosphorylated by CDK6 [87]. MCM proteins are present in great excess at origins of replication and therefore it was postulated that they could have functions independent of their role in DNA replication [88]. MCM proteins were shown to directly bind to HIF1α and inhibit HIF1α and HIF2α transcriptional activity and enhance their proteasomal degradation. In hypoxia, MCM proteins were shown to decrease [86].

HIF1α has been hypothesized to directly inhibit DNA replication independent of its transcriptional activity and its transcriptional partner HIF1β [89]. During hypoxia, HIF1α was shown to bind to Cdc6, which is a critical component necessary for loading the MCM DNA helicase complex onto the DNA. Immunoprecipitation experiments demonstrated that there was an increase in the interaction between Cdc6 and the MCM complex, but that there was a decrease in phosphorylation of the MCM complex by Cdc7. As a consequence of this reduced phosphorylation, replication origin firing and DNA replication were inhibited. The endpoint cellular effect of inhibition of DNA replication, was cell cycle arrest [89]. Although this mechanism was demonstrated for HIF1α, the role of HIF2α in this process was not investigated. It would be interesting to determine if HIF2α could have functionally opposing effects and thus promote DNA replication, in a similar trend to HIFα actions over c-Myc.

## Cell cycle-dependent control HIF

As well as influencing cell cycle progression through its activity as a transcription factor and directly, the levels of HIF1α can also to be influenced by cell cycle regulators. HIF1α has been shown to be subject to a number of post-translational modifications such as phosphorylation, acetylation and sumoylation [90]. A very recent study revealed that HIF1α is phosphorylated by CDK1 on serine 668 [91]. Phosphorylation of HIF1α by CDK1 leads to stabilization of HIF1α levels, even during normoxia. The increased stabilization was associated with an increase in transcription of HIF-dependent target genes, which functionally resulted in enhanced levels of angiogenesis, cell proliferation and invasion. Levels of HIF1α are often associated with poor patient prognosis [92], and taking into account the mechanism described in the study by Warfel and colleagues, this could provide one explanation for the elevation of HIF activity in tumour cells. It would be worthwhile to analyse if a similar modification could be detected on HIF2α. In addition, although HIF1α phosphorylation by CDK1 lead to increase in HIF transcriptional activity, it would be interesting to investigate this modification in the context of the cell cycle-related functions of HIF1α. For example, does phosphorylated HIF1α still induce the expression of p21? Alternatively, can phosphorylated HIF1α still directly inhibit DNA replication? Additional studies should provide answers to these questions.

## The role of PHDs in the cell cycle

The PHD enzymes have long been characterized for their role in regulating HIFα [93]. More recently, new functions for the PHDs have been discovered, which are independent of their ability to directly control HIF. Pyruvate kinase M2 (PKM2), an enzyme crucial in the metabolism of glucose, was found to be hydroxylated by PHD3, on proline 403 and 408. Unlike HIFs, where hydroxylation leads to their ubiquitination and proteasomal degradation, hydroxylation of PKM2 did not result in degradation but did lead to increased interaction between HIF1α and PKM2. Increased interaction between PKM2 and HIF1α, resulted in enhanced HIF1α’s transcriptional activity directed to reprogram cell metabolism [94]. PHDs have been shown to regulate other transcription factors, Activating transcription factor 4 (ATF4) [95, 96]. ATF4 responds to a number of cellular stresses including amino acid depletion, starvation and hypoxia [97]. In addition, it has been shown to be required for tumour cell proliferation under conditions of nutrient depletion [98]. In parallel to the exciting discovery of new substrates for PHDs, there is now some evidence to suggest that PHDs play pivotal roles in the regulation of the cell cycle.

**Fig. 5:**
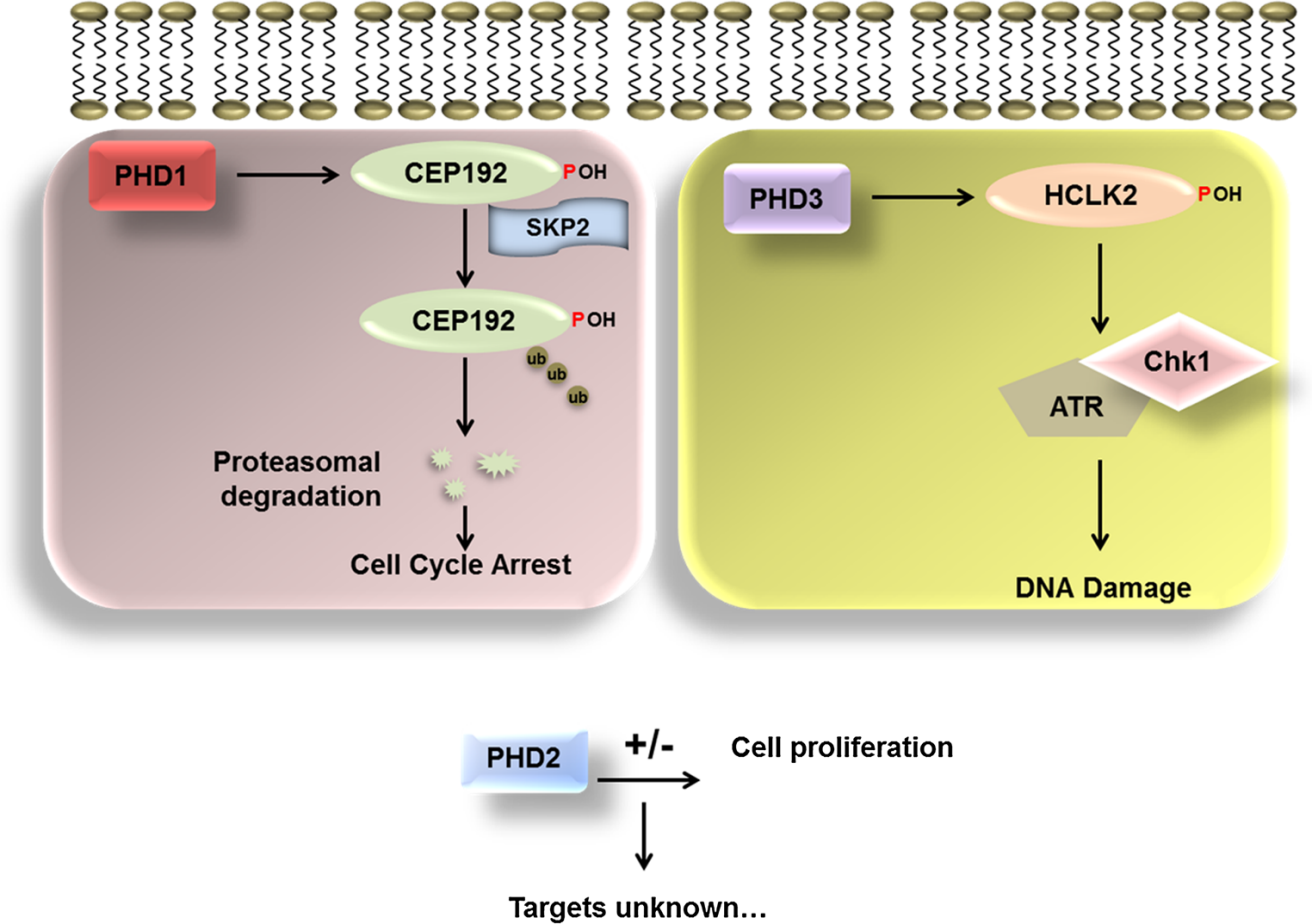
The role of the PHDs in the cell cycle. Schematic diagram showing the roles played by PHD1, 2 and 3 in the cell cycle. PHD1 has been shown to regulate centrosome function and hence mitotic spindle formation through the regulation of the levels of Cep192. Hydroxylation of Cep192 mediates the polyubiquitination by the Skp2 E3 ligase and proteasomal degradation. PHD3 is known to play a key role in the DNA damage response through its ability to hydroxylate HCLK2. Hydroxylation of HCLK2 leads to activation of ATR Chk1 pathway which causes cell cycle arrest. The role of PHD2 in the cell cycle is less clear. Several studies have shown that PHD2 leads to increased proliferation while others have shown a decrease in cell proliferation. Currently, there are no other validated cell cycle targets of PHD2 other than HIFα

PHD3 was shown to regulate, in a hydroxylation-dependent manner, the human clock protein-2 (HCLK2) [99]. HCLK2 is an important protein in the regulation of the mammalian S phase checkpoint. In the absence of HCLK2, cells acquire spontaneous DNA damage during S phase, and impaired recruitment of the DNA damage repair proteins FANCD2 and Rad51 [100]. PHD3 was shown to hydroxylate HCLK2 and this was necessary for HCLK2’s interaction with ATR, which, in turn lead to the activation of ATR/Chk1 and p53. Inhibition of PHD3 activity using the dimethyloxaloylglycine (DMOG), or by hypoxia resulted in a loss of ATR/Chk1 activation. This was compounded by an overall decrease in apoptosis. Through this mechanism, the activity of PHD3 directly controls the DNA damage response and hence cell cycle progression (Fig. 5) [99].

One of the other PHD isoforms, PHD1 has also been strongly linked with the regulation of cell cycle progression. A study by the Kaelin group in 2009 demonstrated a critical role for this protein in the regulation of the levels of cyclin D1 in breast cancer cell lines. Studies in breast cancer cell lines had already shown that PHD1 is oestrogen inducible [101] and in Drosophila, it was shown that there is a genetic interaction between cyclin D1 and PHD1. When PHD1 is depleted using RNA interference, there is a decrease in the total levels of cyclin D1 and, as a consequence, this leads to a decrease in mammary gland proliferation in an in vivo murine model. The effect of PHD1 on cyclin D1 was found to be independent of HIF1α but was dependent on the hydroxylase activity [102]. Importantly, regulation of cyclin D1 was a specific function of PHD1. This study demonstrated a role for PHD1 outside its canonical role as a regulator of HIFs.

More recently, a new substrate for PHD1 was identified in the centrosomal protein Cep192 (Fig. 5) [103]. This study demonstrated that PHD1 was essential for mitotic progression. Loss of PHD1 by RNA interference leads to a mitotic arrest and a mitotic spindle defect, characterized by complete disorganization of the mitotic spindle. Correct mitotic spindle formation has been shown to be dependent on centrosomal duplication and maturation [104]. Cep192 is a large centrosomal protein, which is thought to act as a scaffold protein for the recruitment of other components of the centrosome. Both Cep192 and Cep152 are needed for the recruitment of Polo like kinase 4 (Plk4), a kinase essential for centrosomal duplication [105]. The process of hierarchal recruitment of centrosomal proteins is critical to maintain centriolar number, and is essential to maintain genomic stability. A study by Lee et al. also showed that Cep152 and Cep192 were needed for the recruitment of Plk4, and that loss of either of these proteins resulted in impaired centriole duplication and cell proliferation [106].

PHD1-mediated hydroxylation of Cep192 at proline 1717 results in decreased protein stability. The hydroxylation of Cep192 was shown to be required for the binding of the Skp2 E3 ligase. Binding of Skp2 leads to ubiquitination and degradation of Cep192 by the proteasome [103]. Through this mechanism it is hypothesized that PHD1 regulates the levels of Cep192 on the centrosome‚ and should the cell encounter a stress such as hypoxia, the resulting inhibition of PHD1 function, would lead to cell cycle arrest. This mechanism was shown to be HIF independent.

PHDs have also been shown to be important sensors of amino acids and play a role in mTOR signaling [107]. It is thus possible that changes in amino acids, or additional metabolites derived from the Krebs cycle, could result in similar changes to Cep192 as hypoxia. However, this requires additional research. It would also be of interest to determine if hydroxylation by PHDs plays a wider role in cell cycle regulation, and also if the PHDs themselves are subject to cell cycle regulation.

Thus far, there are no new PHD2 substrates, apart from HIFα, that have any connection with the cell cycle. However, several studies have shown that PHD2 levels can change the proliferative potential in a cell type-specific manner (Fig. 5). As such, reduction of PHD2 levels in cancer cells leads to increase tumour growth due to increased proliferation of endothelial cells [108]. Similarly, mutations in PHD2 have been associated with paragangliomas [109]. However, deletion of PHD2 in myeloid cells has been shown to result in tumour suppression [110]. Also, reduction of PHD2 levels by RNA interference has been shown to reduce breast cancer cell proliferation [111]. All of these studies, despite having opposing outcomes, highlighted the importance of PHD2. Whether PHD2 has additional substrates, with closer links to the cell cycle machinery would require additional studies.

## The role of VHL and mitosis

In addition to the studies described above, linking both the HIFs and PHDs to the modulation of cell cycle control, one other key player in the cellular response to hypoxia has also been studied for its role in mitosis, von Hippel–Lindau (VHL) protein. VHL is part of the E3 ligase which targets HIF to the proteasome following PHD-mediated hydroxylation [27]. Mutations in VHL are often associated with a predisposition to kidney cancer [112]. A potential cause for the cancer predisposition in VHL patients is due to misregulation of the HIFs, as both HIF1α and HIF2α have been found to correlate with poor patient prognosis [92, 113]. However, more recently, VHL has also been shown to regulate mitotic spindle. In a study by Krek and colleagues, it was shown that VHL localizes to mitotic spindle in human cells [114]. Loss of function of VHL was shown to lead to spindle and spindle checkpoint defects that ultimately control chromosome instability. The defects in mitotic spindle were attributed to unstable microtubules [114]. Astral microtubules have been shown to be required for mitotic progression, as they are critical for the correct positioning and orientation of the mitotic spindle [115].

Defects in the spindle checkpoint were due to reduced levels of mitotic arrest deficient 2 (Mad2), a protein critical for normal spindle checkpoint function [116]. This suggests that, as well as regulating astral microtubules, VHL also plays an essential role in regulating the spindle checkpoint. The role of VHL in regulating mitotic spindle and checkpoint function was further supported by more recent work, where it was demonstrated that VHL is a novel substrate for Aurora A phosphorylation [117]. Aurora A is a cell cycle regulated kinase, whose expression is regulated through proteasomal degradation. Aurora A is involved in duplication of centrosomes, correct spindle formation and stability [118]. VHL is phosphorylated at serine 72 by Aurora A, in vivo [117]. Future work should determine what the functional significance of VHL phosphorylation at this site. For example, how does it affect spindle formation and the checkpoint response, and also its connection with VHL action over the HIFα isoforms.

## Additional cellular oxygen sensors and the cell cycle

In addition to the PHDs and the HIFs, the cell possesses other proteins that are also oxygen sensors, these include FIH and the structural related Jumoji C containing (JMJC) family of demethylases [119].

### FIH

Unlike the PHD, FIH has been shown to have many additional substrates to HIF [120]. Although the physiological relevance of the FIH-dependent hydroxylation for many of these is still unknown, FIH has several important targets such as Notch, ASPP2, and NF-κB subunits. FIH has good affinity for Ankyrin repeat containing proteins [121]. Despite these important substrates, functional effects of FIH on the cell cycle are not wide reported. One study has reported that FIH can impact of p53 function, with FIH depletion inducing p53 and its target gene p21, resulting in cell cycle arrest [122]. In addition, reduction of FIH via depletion of the chromatin remodeler ISWI also resulted in cell cycle arrest and reduced proliferation [123]. Despite these two studies, FIH links to the cell cycle remain tenuous.

### JmjC

**Fig. 6:**
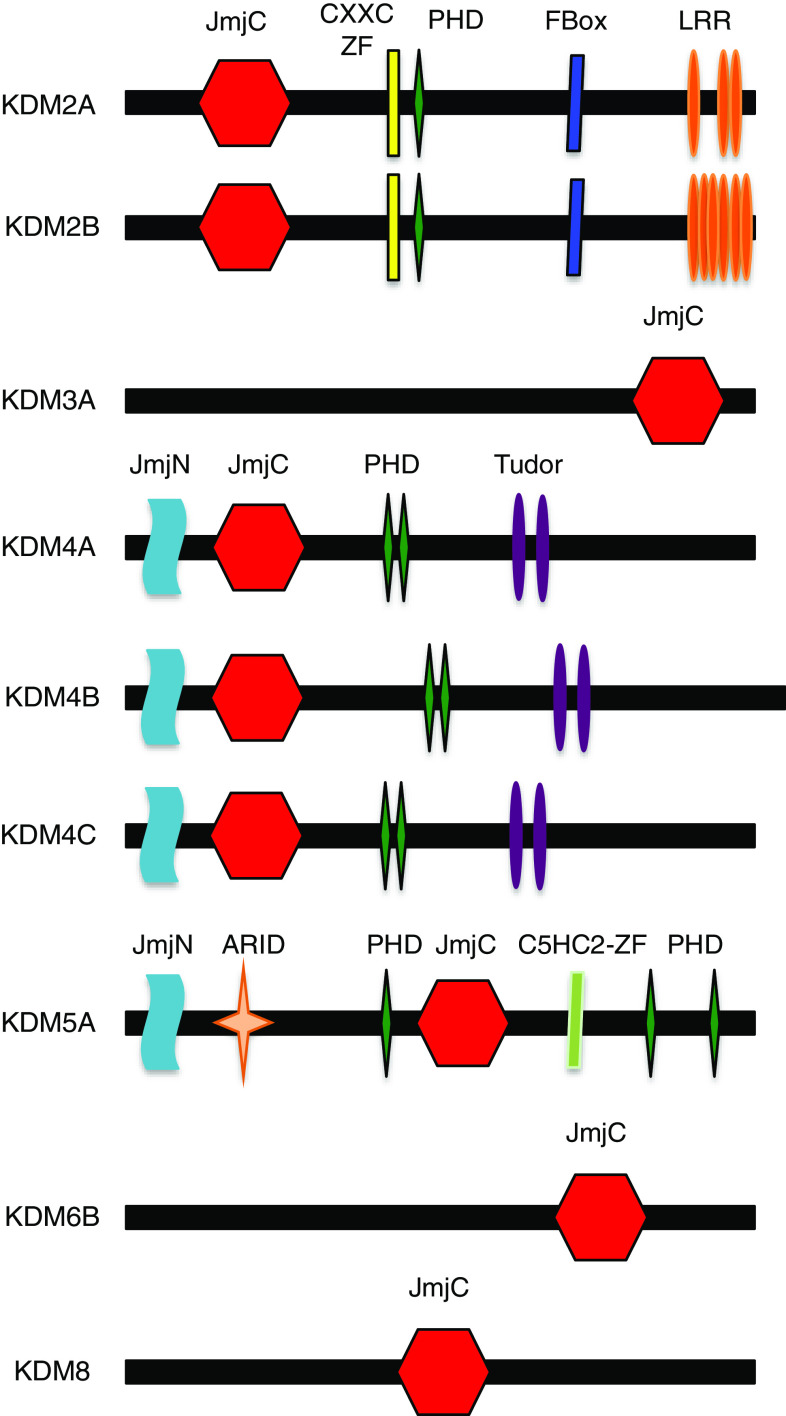
JmJC enzymes with roles in the cell cycle. Schematic diagram showing the domain structure of several of the JmJC enzymes. Depicted are JmJC enzymes with roles in controlling cell cycle progression. Domains present in these enzymes are: *JmJC* Jumoni C, *CXXC-ZF* CXXC zinc finger, *PHD* plant homeodomain, F-box; *LRR* leucine rich repeat; Tudor; *ARID* AT-rich interacting domain, *C5HC2-ZF* C5HC2 zinc finger

The JmjC proteins catalyse histone, protein and DNA demethylation through a hydroxylation reaction. The catalytic activity of these enzymes depends on Fe^2+^ and α-KG for demethylating mono-, di- and tri-methylated residues [124]. Interestingly, the Jumonji (JmJC) domain is found in the asparagine hydroxylase FIH, a known regulator of HIF activity (Fig. 6). The JmJC demethylases are therefore dioxygenases that use molecular oxygen and α-KG as cofactors [125].

Chromatin is a highly complex structure composed of protein and DNA. The basic unit of chromatin is the nucleosome, composed of 147 bp of DNA wrapped around an octamer of histones [126]. Different levels of compaction and organization occur in different regions in the nucleus, as well as, with the stage of the cell cycle [127]. Histone tail modification can lead to changes in chromatin structure, which have functional consequences for the several processes occurring in the nucleus [124, 128]. For example, methylation on arginine or lysine residues can have repressive or activating consequences on gene expression depending not only, on which particular residue which is modified but also, on how many methyl groups are added [129, 130]. Lysine methylation on histones is thus an important post-translational modification that can result in changes in chromatin structure. Deregulation of this modification has been associated with several human cancers [131]. Histone methyltransferases and demethylases thus play role in balancing methylation dynamics.

Tumours often develop hypoxic regions, which control survival and proliferation of cells. The majority of the JmJC demethylases is hypoxia inducible [132, 133]. Moreover some of these enzymes are direct targets of HIF and have been shown to contribute to the cellular response to hypoxia. Deregulation of histone demethylation can result in perturbation of the cell cycle. For example fumarate hydratase (FH) and succinate dehydrogenase (SDH) are two Krebs cycle enzymes found mutated in a variety of human cancers. Accumulation of their products, fumarate and succinate, inhibits the α-KG-dioxygenases including JmJC histone demethylases and PHDs with an associated increases in histone and DNA methylation and HIF1α accumulation [134], which is thought to contribute to the cancer phenotype.

While some histone demethylases have defined substrate specificity, others share a variety of substrates, and therefore display redundancy in regulating chromatin and gene expression. Several studies have described that JmJC demethylase activity, with the expression or repression of genes, is involved in the regulation of the cell cycle. Some of these are reviewed below.

The JmJC domain containing histone demethylase 1 (KDM2A) gene encodes a member of the F-box protein family (Fig. 6) and demethylase with activity towards lysine 36 of histone 3 (H3K36) [135]. It demethylases H3K36 via a hydroxylation-based mechanism [125]. KDM2A gene is induced under hypoxia conditions [132, 133] but the mechanism and functional significance toward the hypoxia response is currently not known. KDM2A is frequently overexpressed in non-small cell lung cancer (NSCLC) tumours and cell lines [136]. Demethylation of H3K36(2me) at the dual specificity phosphatase 3 (DUSP3) promoter by KDM2A inhibits DUSP3 gene transcription [136]. The consequence of this repression are an increase in ERK1/2 phosphorylation levels in NSCLC cells, thus contributing to cell proliferation and invasiveness [136]. Methylated H3K36 is often present in regions associated with active transcription [137]. This mark has also been linked to transcriptional elongation [138]. Furthermore, it has been reported that the histone mark, H3K36me3, is mostly present in the coding regions of active genes [139].

Recently, it has been shown that the histone mark H3K36me3 is required in G1 and early S phases to ensure DNA mismatch repair before DNA replication [140]. Moreover, KDM2A, and its demethylase activity, has also been shown to sustain centromeric integrity and genomic stability during mitosis [141]. In addition, depletion of KDM2A inhibits mesenchymal stem cell proliferation and arrests the cell cycle progression at G1/S phase. This cell cycle arrest occurs via the derepression of cyclin-dependent kinase inhibitors, p15 and p27 [142]. However, overexpression of specific KDM2A spliced variant improves the proliferation of human embryonic stem cells derived keratinocytes [143]. KDM2B (Fig. 6) has also been implicated in the control of the cell cycle via its role in the regulation of p15 [144].

Another example, histone demethylation in cell cycle regulation involves KDM3A (Fig. 6) that has an important role in human carcinogenesis. In cancer cells, KDM3A knockdown promotes cell cycle arrest, whereas elevated KDM3A levels promote G1/S transition. KDM3A activates the *HOXA1* gene expression through demethylation of H3K9me2 [145]. Increased HOXA1 activates Cyclin D1 gene transcription, which actively promotes the transition from G1 to S, hence resulting in cancer cells proliferation. Similarly, regulation of KDM3A by HIF1α enhances hypoxic gene expression and tumour growth [146].

KDM4/JMJD2 (Fig. 6) is a family of demethylases with a tumorigenic role and is overexpressed in several cancers such as, breast, colorectal, lung, and prostate [147]. KDM4A regulates cell cycle progression and DNA replication and these roles depend on its enzymatic activity. Demethylation of H3K9me3 by KDM4A (or its nematode ortholog JMJD-2) regulates DNA replication by antagonizing HP1γ and controlling chromatin accessibility [148]. Furthermore, KDM4A protein levels are regulated by the 26S proteasome in a cell cycle-dependent manner, with highest expression in G1/S and decreasing in G2/M [148, 149].

Another member of KDM4 family is KDM4B (JMJD2B). KDM4B demethylase activity is upregulated in bladder and lung cancer cells, and it has been shown to regulate the G1-S phase transition of the cell cycle. Knockdown of KDM4B inhibits cell proliferation, leading to a decrease of cells in S phase, and an increase of cells population in G1. KDM4B induces CDK6 expression through demethylation of H3K9me3 at the CDK6 promoter [150]. In addition, KDM4B mediates gastric cancer cells proliferation under hypoxia by stimulating cyclin A1 gene expression. Hypoxia upregulates KDM4B expression, which demethylates H3K9 at Cyclin A1 promoter, stimulating gastric cancer cell proliferation. Moreover, radiation-mediated reduction in KDM4B decreases CCNA1 expression, which resulted in a decrease of cancer gastric cells growth [151]. More recently, it has been shown that the knockdown of KDM4B in colorectal cancer cells mediates STAT3 suppression, which in turn activates the DNA damage response and cell cycle arrest [152].

The KDM4C (JMJD2C) promotes breast cancer cell transformation and proliferation [153]. Furthermore, KDM4C has been described as a HIF1α co-activator. KDM4C was shown to demethylate H3K9me3 at HREs of HIF1α target genes, therefore increasing HIF1α binding. Hence, KDM4C simulates HIF1α-mediated transactivation of genes that are involved in breast cancer progression [154].

 KDM6B (JMJD3) (Fig. 6) is another demethylase with links to cell cycle [155]. KDM6B demethylates H3K27me3, a very important repressive mark on chromatin [156]. Specifically linked to cell cycle, KDM6B has been shown to be required for the demethylation of the INK4/ARF locus in response to oncogene activation [155, 156]. The INK4/ARF locus expresses two very important cell cycle regulators: p14ARF and p16IKN4, which are crucial for cell cycle arrest induction [157].

JMJD5 (KDM8) (Fig. 6) has also been shown to regulate cell proliferation [158, 159]. JMJD5 demethylases H3K36me2 [158]. It has been shown to control both cyclin A1 expression [158] and p21 [159]. It has also been suggested to act as a tumour suppressor [160].

As more of the JMJC-containing enzymes are investigated, more detailed information linking them to cell cycle is possible. In addition, their dependency to oxygen levels and additional control mechanisms are yet to be fully understood. The next years will therefore be very informative.

## Conclusions

Sensing and responding to hypoxia (low oxygen) are important for many physiological processes, of which cell cycle is a very important one. As such, cells respond to changes in oxygen availability by altering the activity of a variety of oxygen sensors in the cell and activating a specific transcriptional program. Of these, HIFs, PHD and more recently JmjC have important roles to play. While the role of HIF in the control of the cell cycle has been described by many studies, more recent studies have uncovered roles for individual PHDs and JmjC proteins in direct or indirect control of the cell cycle machinery. As such, the cell has equipped itself with many possible control mechanisms to prevent catastrophe from happening at any stage of the cell cycle, when oxygen levels drop below the acceptable. With the recent finding reviewed here, more exciting and fascinating insights into the cell safeguard mechanism when dealing with low oxygen will most definitely be discovered in the near future.

## References

[1] Kenneth NS, Rocha S (2008). Regulation of gene expression by hypoxia. Biochem J.

[2] Wang GL (1995). Hypoxia-inducible factor 1 is a basic-helix-loop-helix-PAS heterodimer regulated by cellular O_2_ tension. Proc Natl Acad Sci.

[3] Hu C-J (2003). Differential roles of hypoxia-inducible factor 1α (HIF-1α) and HIF-2α in hypoxic gene regulation. Mol Cell Biol.

[4] Huang LE (1998). Regulation of hypoxia-inducible factor 1α is mediated by an O_2_-dependent degradation domain via the ubiquitin-proteasome pathway. Proc Natl Acad Sci.

[5] Makino Y (2001). Inhibitory PAS domain protein is a negative regulator of hypoxia-inducible gene expression. Nature.

[6] Zhang P (2014). Hypoxia-inducible factor 3 is an oxygen-dependent transcription activator and regulates a distinct transcriptional response to hypoxia. Cell Rep.

[7] Hu C-J (2006). Differential regulation of the transcriptional activities of hypoxia-inducible factor 1 alpha (HIF-1α) and HIF-2α in stem cells. Mol Cell Biol.

[8] Hubbi ME (2013). Chaperone-mediated autophagy targets hypoxia-inducible factor-1α (HIF-1α) for lysosomal degradation. J Biol Chem.

[9] van Uden P (2011). Evolutionary conserved regulation of HIF-1β by NF-κB. PLoS Genet.

[10] Rius J (2008). NF-[kgr]B links innate immunity to the hypoxic response through transcriptional regulation of HIF-1[agr]. Nature.

[11] Hudson CC (2002). Regulation of hypoxia-inducible factor 1α expression and function by the mammalian target of rapamycin. Mol Cell Biol.

[12] Toschi A (2008). Differential dependence of hypoxia-inducible factors 1α and 2α on mTORC1 and mTORC2. J Biol Chem.

[13] Bett JS (2013). The P-body component USP52/PAN2 is a novel regulator of HIF1A mRNA stability. Biochem J.

[14] Kaelin WG, Ratcliffe PJ (2008). Oxygen sensing by metazoans: the central role of the HIF hydroxylase pathway. Mol Cell.

[15] Haase VH (2012). Renal cancer: oxygen meets metabolism. Exp Cell Res.

[16] Appelhoff RJ (2004). Differential function of the prolyl hydroxylases PHD1, PHD2, and PHD3 in the regulation of hypoxia-inducible factor. J Biol Chem.

[17] Berra E (2003). HIF prolyl-hydroxylase 2 is the key oxygen sensor setting low steady-state levels of HIF-1α in normoxia. EMBO J.

[18] Epstein ACR (2001). *C. elegans* EGL-9 and mammalian homologs define a family of dioxygenases that regulate HIF by prolyl hydroxylation. Cell.

[19] Fandrey J, Gorr TA, Gassmann M (2006). Regulating cellular oxygen sensing by hydroxylation. Cardiovasc Res.

[20] Semenza GL (2003). Targeting HIF-1 for cancer therapy. Nat Rev Cancer.

[21] Jaakkola P (2001). Targeting of HIF-α to the von Hippel–Lindau ubiquitylation complex by O_2_-regulated prolyl hydroxylation. Science.

[22] McCahill A (2002). The RACK1 scaffold protein: a dynamic cog in cell response mechanisms. Mol Pharmacol.

[23] Liu YV (2007). RACK1 competes with HSP90 for binding to HIF-1α and is required for O_2_-independent and HSP90 inhibitor-induced degradation of HIF-1α. Mol Cell.

[24] Semenza GL (1996). Hypoxia response elements in the aldolase A, enolase 1, and lactate dehydrogenase A gene promoters contain essential binding sites for hypoxia-inducible factor 1. J Biol Chem.

[25] Schödel J (2011). High-resolution genome-wide mapping of HIF-binding sites by ChIP-seq. Blood.

[26] Lando D (2002). FIH-1 is an asparaginyl hydroxylase enzyme that regulates the transcriptional activity of hypoxia-inducible factor. Genes Dev.

[27] Rocha S (2007). Gene regulation under low oxygen: holding your breath for transcription. Trends Biochem Sci.

[28] Masson N (2012). The FIH hydroxylase is a cellular peroxide sensor that modulates HIF transcriptional activity. EMBO Rep.

[29] Tracy K (2007). BNIP3 is an RB/E2F target gene required for hypoxia-induced autophagy. Mol Cell Biol.

[30] Kim J-W (2006). HIF-1-mediated expression of pyruvate dehydrogenase kinase: a metabolic switch required for cellular adaptation to hypoxia. Cell Metab.

[31] Bi M (2005). ER stress-regulated translation increases tolerance to extreme hypoxia and promotes tumor growth. EMBO J.

[32] Liu L (2006). Hypoxia-induced energy stress regulates mRNA translation and cell growth. Mol Cell.

[33] Semenza GL (2001). Hypoxia-inducible factor 1: oxygen homeostasis and disease pathophysiology. Trends Mol Med.

[34] Haase VH (2006). The VHL//HIF oxygen-sensing pathway and its relevance to kidney disease. Kidney Int.

[35] Liu L (2008). Hypoxia-inducible factor-1α contributes to hypoxia-induced chemoresistance in gastric cancer. Cancer Sci.

[36] Bertoli C, Skotheim JM, de Bruin RAM (2013). Control of cell cycle transcription during G1 and S phases. Nat Rev Mol Cell Biol.

[37] Swann MM (1957). The control of cell division: a review: I. General mechanisms. Cancer Res.

[38] Sengupta T (2011). Hypoxia-inducible factor 1 is activated by dysregulated cyclin E during mammary epithelial morphogenesis. Mol Cell Biol.

[39] Graña X, Garriga J, Mayol X (1998). Role of the retinoblastoma protein family, pRB, p107 and p130 in the negative control of cell growth. Oncogene.

[40] Classon M, Harlow E (2002). The retinoblastoma tumour suppressor in development and cancer. Nat Rev Cancer.

[41] Krtolica A, Krucher NA, Ludlow JW (1998). Hypoxia-induced pRB hypophosphorylation results from downregulation of CDK and upregulation of PP1 activities. Oncogene.

[42] Weinberg RA (1995). The retinoblastoma protein and cell cycle control. Cell.

[43] Simanis V, Nurse P (1986). The cell cycle control gene cdc2+ of fission yeast encodes a protein kinase potentially regulated by phosphorylation. Cell.

[44] Pavletich NP (1999). Mechanisms of cyclin-dependent kinase regulation: structures of cdks, their cyclin activators, and cip and INK4 inhibitors. J Mol Biol.

[45] Lolli G, Johnson LN (2005). CAK—cyclin-dependent activating kinase: a key kinase in cell cycle control and a target for drugs?. Cell Cycle.

[46] Russell P, Nurse P (1987). Negative regulation of mitosis by wee1+, a gene encoding a protein kinase homolog. Cell.

[47] Donzelli M, Draetta GF (2003). Regulating mammalian checkpoints through Cdc25 inactivation. EMBO Rep.

[48] Toyoshima H, Hunter T (1994). p27, a novel inhibitor of G1 cyclin-Cdk protein kinase activity, is related to p21. Cell.

[49] Aprelikova O, Xiong Y, Liu ET (1995). Both p16 and p21 families of cyclin-dependent kinase (CDK) inhibitors block the phosphorylation of cyclin-dependent kinases by the CDK-activating kinase. J Biol Chem.

[50] Afshari CA (1996). A role for a p21-E2F interaction during senescence arrest of normal human fibroblasts. Cell Growth Differ: Mol Biol J Am Assoc Cancer Res.

[51] Li Y (1994). Cell cycle expression and p53 regulation of the cyclin-dependent kinase inhibitor p21. Oncogene.

[52] Yamada K (2013). Identification and functional characterization of FMN2, a regulator of the cyclin-dependent kinase inhibitor p21. Mol Cell.

[53] Malumbres M, Barbacid M (2001). Milestones in cell division: to cycle or not to cycle: a critical decision in cancer. Nat Rev Cancer.

[54] Mill P (2005). Shh controls epithelial proliferation via independent pathways that converge on N-Myc. Dev Cell.

[55] Dang CV (2008). The interplay between MYC and HIF in cancer. Nat Rev Cancer.

[56] Adhikary S, Eilers M (2005). Transcriptional regulation and transformation by Myc proteins. Nat Rev Mol Cell Biol.

[57] Gartel AL (2001). Myc represses the p21(WAF1/CIP1) promoter and interacts with Sp1/Sp3. Proc Natl Acad Sci.

[58] Mateyak MK, Obaya AJ, Sedivy JM (1999). c-Myc regulates cyclin D-Cdk4 and -Cdk6 activity but affects cell cycle progression at multiple independent points. Mol Cell Biol.

[59] Wu L (2001). The E2F1-3 transcription factors are essential for cellular proliferation. Nature.

[60] Moniz S, Biddlestone J, Rocha S (2014). Grow_2_: the HIF system, energy homeostasis and the cell cycle. Histol Histopathol.

[61] Iida T (2002). Hypoxia-inducible factor-1α induces cell cycle arrest of endothelial cells. Genes Cells.

[62] Koshiji M (2004). HIF-1α induces cell cycle arrest by functionally counteracting Myc. EMBO J.

[63] Hackenbeck T (2009). HIF-1 or HIF-2 induction is sufficient to achieve cell cycle arrest in NIH3T3 mouse fibroblasts independent from hypoxia. Cell Cycle.

[64] Vietri M (2006). Direct interaction between the catalytic subunit of protein phosphatase 1 and pRb. Cancer Cell Int.

[65] Goda N (2003). Hypoxia-inducible factor 1α is essential for cell cycle arrest during hypoxia. Mol Cell Biol.

[66] Lim J-H (2006). Bafilomycin induces the p21-mediated growth inhibition of cancer cells under hypoxic conditions by expressing hypoxia-inducible factor-1α. Mol Pharmacol.

[67] Gardner LB (2001). Hypoxia inhibits G1/S transition through regulation of p27 expression. J Biol Chem.

[68] Culver C (2011). HIF-1α depletion results in SP1-mediated cell cycle disruption and alters the cellular response to chemotherapeutic drugs. Cell Cycle.

[69] Bouchard C (1999). Direct induction of cyclin D2 by Myc contributes to cell cycle progression and sequestration of p27. EMBO J.

[70] Yang W (2001). Repression of transcription of the p27 Kip1 cyclin-dependent kinase inhibitor gene by c-Myc. Oncogene.

[71] Gordan JD (2007). HIF-2α promotes hypoxic cell proliferation by enhancing c-myc transcriptional activity. Cancer Cell.

[72] Covello KL (2006). HIF-2α regulates Oct-4: effects of hypoxia on stem cell function, embryonic development, and tumor growth. Genes Dev.

[73] Elorza A (2012). HIF2α acts as an mTORC1 activator through the amino acid carrier SLC7A5. Mol Cell.

[74] Kulshreshtha R (2007). A MicroRNA signature of hypoxia. Mol Cell Biol.

[75] Chan SY (2009). MicroRNA-210 controls mitochondrial metabolism during hypoxia by repressing the iron–sulfur cluster assembly proteins ISCU1/2. Cell Metab.

[76] Medina PP, Slack FJ (2008). microRNAs and cancer. Cell Cycle.

[77] Huang X (2009). Hypoxia-inducible mir-210 regulates normoxic gene expression involved in tumor initiation. Mol Cell.

[78] Gee HE (2010). hsa-miR-210 is a marker of tumor hypoxia and a prognostic factor in head and neck cancer. Cancer.

[79] Giannakakis A (2008). miR-210 links hypoxia with cell cycle regulation and is deleted in human epithelial ovarian cancer. Cancer Biol Ther.

[80] Zhang Z (2009). MicroRNA miR-210 modulates cellular response to hypoxia through the MYC antagonist MNT. Cell Cycle.

[81] Young SD, Marshall RS, Hill RP (1988). Hypoxia induces DNA overreplication and enhances metastatic potential of murine tumor cells. Proc Natl Acad Sci.

[82] Hammond EM, Dorie MJ, Giaccia AJ (2003). ATR/ATM targets are phosphorylated by ATR in response to hypoxia and ATM in response to reoxygenation. J Biol Chem.

[83] Ding G (2013). HIF1-regulated ATRIP expression is required for hypoxia induced ATR activation. FEBS Lett.

[84] Kastan MB, Bartek J (2004). Cell-cycle checkpoints and cancer. Nature.

[85] Hammond EM, Green SL, Giaccia AJ (2003). Comparison of hypoxia-induced replication arrest with hydroxyurea and aphidicolin-induced arrest. Mut Res/Fundam Mol Mech Mutagen.

[86] Hubbi ME (2011). MCM proteins are negative regulators of hypoxia-inducible factor 1. Mol Cell.

[87] Sclafani RA, Holzen TM (2007). Cell cycle regulation of DNA replication. Annu Rev Genet.

[88] Blow JJ, Hodgson B (2002). Replication licensing—origin licensing: defining the proliferative state?. Trends Cell Biol.

[89] Hubbi ME (2013). A nontranscriptional role for HIF-1{alpha} as a direct inhibitor of DNA replication. Sci Signal.

[90] Cheng J (2007). SUMO-specific protease 1 is essential for stabilization of HIF1α during hypoxia. Cell.

[91] Warfel NA (2013). CDK1 stabilizes HIF-1alpha via direct phosphorylation of Ser668 to promote tumor growth. Cell Cycle.

[92] Birner P (2000). Overexpression of hypoxia-inducible factor 1α is a marker for an unfavorable prognosis in early-stage invasive cervical cancer. Cancer Res.

[93] Schofield CJ, Ratcliffe PJ (2004). Oxygen sensing by HIF hydroxylases. Nat Rev Mol Cell Biol.

[94] Luo W (2011). Pyruvate kinase M2 is a PHD3-stimulated coactivator for hypoxia-inducible factor 1. Cell.

[95] Köditz J (2007). Oxygen-dependent ATF-4 stability is mediated by the PHD3 oxygen sensor. Blood.

[96] Hiwatashi Y (2011). PHD1 interacts with ATF4 and negatively regulates its transcriptional activity without prolyl hydroxylation. Exp Cell Res.

[97] Rutkowski DT, Kaufman RJ (2003). All roads lead to ATF4. Dev Cell.

[98] Ye J (2010). The GCN2-ATF4 pathway is critical for tumour cell survival and proliferation in response to nutrient deprivation. EMBO J.

[99] Xie L (2012). PHD3-dependent hydroxylation of HCLK2 promotes the DNA damage response. J Clin Invest.

[100] Collis SJ (2007). HCLK2 is essential for the mammalian S-phase checkpoint and impacts on Chk1 stability. Nat Cell Biol.

[101] Seth P (2002). Novel estrogen and tamoxifen induced genes identified by SAGE (serial analysis of gene expression). Oncogene.

[102] Zhang Q (2009). Control of cyclin D1 and breast tumorigenesis by the EglN2 prolyl hydroxylase. Cancer Cell.

[103] Moser SC (2013). PHD1 links cell-cycle progression to oxygen sensing through hydroxylation of the centrosomal protein Cep192. Dev Cell.

[104] Joukov V (2010). Centrosomal protein of 192 kDa (Cep192) promotes centrosome-driven spindle assembly by engaging in organelle-specific Aurora A activation. Proc Natl Acad Sci USA.

[105] Sonnen KF (2013). Human Cep192 and Cep152 cooperate in Plk4 recruitment and centriole duplication. J Cell Sci.

[106] Kim TS (2013). Hierarchical recruitment of Plk4 and regulation of centriole biogenesis by two centrosomal scaffolds, Cep192 and Cep152. Proc Natl Acad Sci USA.

[107] Duran RV (2013). HIF-independent role of prolyl hydroxylases in the cellular response to amino acids. Oncogene.

[108] Chan DA (2009). Tumor vasculature is regulated by PHD2-mediated angiogenesis and bone marrow-derived cell recruitment. Cancer Cell.

[109] Ladroue C (2008). PHD2 mutation and congenital erythrocytosis with paraganglioma. N Engl J Med.

[110] Mamlouk S (2014). Loss of prolyl hydroxylase-2 in myeloid cells and T-lymphocytes impairs tumor development. Int J Cancer.

[111] Wottawa M (2013). Knockdown of prolyl-4-hydroxylase domain 2 inhibits tumor growth of human breast cancer MDA-MB-231 cells by affecting TGF-β1 processing. Int J Cancer.

[112] Gnarra I (1994). Mutations of the VHL tumour suppressor gene in renal. Nat Genet.

[113] Giatromanolaki A (2003). Hypoxia-inducible factors 1α and 2α are related to vascular endothelial growth factor expression and a poorer prognosis in nodular malignant melanomas of the skin. Melanoma Res.

[114] Thoma CR (2009). VHL loss causes spindle misorientation and chromosome instability. Nat Cell Biol.

[115] Wittmann T, Hyman A, Desai A (2001). The spindle: a dynamic assembly of microtubules and motors. Nat Cell Biol.

[116] Schuyler SC, Wu YF, Kuan VJ (2012). The Mad1-Mad2 balancing act—a damaged spindle checkpoint in chromosome instability and cancer. J Cell Sci.

[117] Martin B (2013). Identification of pVHL as a novel substrate for Aurora-A in clear cell renal cell carcinoma (ccRCC). PLoS One.

[118] Nikonova AS (2013). Aurora A kinase (AURKA) in normal and pathological cell division. Cell Mol Life Sci.

[119] Chen Z (2006). Structural insights into histone demethylation by JMJD2 family members. Cell.

[120] Cockman ME (2009). Proteomics-based identification of novel factor inhibiting hypoxia-inducible factor (FIH) substrates indicates widespread asparaginyl hydroxylation of ankyrin repeat domain-containing proteins. Mol Cell Proteomics.

[121] Cockman ME, Webb JD, Ratcliffe PJ (2009). FIH-dependent asparaginyl hydroxylation of ankyrin repeat domain-containing proteins. Ann N Y Acad Sci.

[122] Pelletier J (2011). The asparaginyl hydroxylase factor-inhibiting HIF is essential for tumor growth through suppression of the p53–p21 axis. Oncogene.

[123] Melvin A, Mudie S, Rocha S (2011). The chromatin remodeler ISWI regulates the cellular response to hypoxia: role of FIH. Mol Biol Cell.

[124] Kooistra SM, Helin K (2012). Molecular mechanisms and potential functions of histone demethylases. Nat Rev Mol Cell Biol.

[125] Tsukada Y-I (2012). Hydroxylation mediates chromatin demethylation. J Biochem.

[126] Hayes JJ, Hansen JC (2001). Nucleosomes and the chromatin fiber. Curr Opin Genet Dev.

[127] Llères D (2009). Quantitative analysis of chromatin compaction in living cells using FLIM–FRET. J Cell Biol.

[128] Cedar H, Bergman Y (2009). Linking DNA methylation and histone modification: patterns and paradigms. Nat Rev Genet.

[129] Martin C, Zhang Y (2005). The diverse functions of histone lysine methylation. Nat Rev Mol Cell Biol.

[130] Santos-Rosa H (2002). Active genes are tri-methylated at K4 of histone H3. Nature.

[131] Varier RA, Timmers H (2011). Histone lysine methylation and demethylation pathways in cancer. Biochim Biophys Acta (BBA)—Rev Cancer.

[132] Xia X (2009). Integrative analysis of HIF binding and transactivation reveals its role in maintaining histone methylation homeostasis. Proc Natl Acad Sci.

[133] Melvin A, Rocha S (2012). Chromatin as an oxygen sensor and active player in the hypoxia response. Cell Signal.

[134] Xiao M (2012). Inhibition of α-KG-dependent histone and DNA demethylases by fumarate and succinate that are accumulated in mutations of FH and SDH tumor suppressors. Genes Dev.

[135] Tsukada Y-I (2006). Histone demethylation by a family of JmjC domain-containing proteins. Nature.

[136] Wagner KW (2013). KDM2A promotes lung tumorigenesis by epigenetically enhancing ERK1/2 signaling. J Clin Investig.

[137] Pokholok DK (2005). Genome-wide map of nucleosome acetylation and methylation in yeast. Cell.

[138] Xiao T (2003). Phosphorylation of RNA polymerase II CTD regulates H3 methylation in yeast. Genes Dev.

[139] Mikkelsen TS (2007). Genome-wide maps of chromatin state in pluripotent and lineage-committed cells. Nature.

[140] Li F (2013). The histone Mark H3K36me3 regulates human DNA mismatch repair through its interaction with MutSα. Cell.

[141] Frescas D (2008). KDM2A represses transcription of centromeric satellite repeats and maintains the heterochromatic state. Cell Cycle.

[142] Gao R (2013). Depletion of histone demethylase KDM2A inhibited cell proliferation of stem cells from apical papilla by de-repression of p15INK4B and p27Kip1. Mol Cell Biochem.

[143] Iuchi S, Green H (2012). Lysine-specific demethylase 2A (KDM2A) normalizes human embryonic stem cell derived keratinocytes. Proc Natl Acad Sci.

[144] He J (2008). The H3K36 demethylase Jhdm1b/Kdm2b regulates cell proliferation and senescence through p15Ink4b. Nat Struct Mol Biol.

[145] Cho HS (2012). The JmjC domain-containing histone demethylase KDM3A is a positive regulator of the G1/S transition in cancer cells via transcriptional regulation of the HOXA1 gene. Int J Cancer.

[146] Krieg AJ (2010). Regulation of the histone demethylase JMJD1A by hypoxia-inducible factor 1α enhances hypoxic gene expression and tumor growth. Mol Cell Biol.

[147] Berry WL, Janknecht R (2013). KDM4/JMJD2 histone demethylases: epigenetic regulators in cancer cells. Cancer Res.

[148] Black JC (2010). Conserved antagonism between JMJD2A/KDM4A and HP1γ during cell cycle progression. Mol Cell.

[149] Van Rechem C (2011). The SKP1-Cul1-F-box and leucine-rich repeat protein 4 (SCF-FbxL4) ubiquitin ligase regulates lysine demethylase 4A (KDM4A)/Jumonji domain-containing 2A (JMJD2A) protein. J Biol Chem.

[150] Toyokawa G (2011). The histone demethylase JMJD2B plays an essential role in human carcinogenesis through positive regulation of cyclin-dependent kinase 6. Cancer Prev Res.

[151] Kim J-G (2012). Histone demethylase JMJD2B-mediated cell proliferation regulated by hypoxia and radiation in gastric cancer cell. Biochim Biophys Acta (BBA)—Gene Regul Mech.

[152] Chen L (2014). Jumonji domain-containing protein 2B silencing induces DNA damage response via STAT3 pathway in colorectal cancer. Br J Cancer.

[153] Liu G (2009). Genomic amplification and oncogenic properties of the GASC1 histone demethylase gene in breast cancer. Oncogene.

[154] Luo W (2012). Histone demethylase JMJD2C is a coactivator for hypoxia-inducible factor 1 that is required for breast cancer progression. Proc Natl Acad Sci.

[155] Barradas M (2009). Histone demethylase JMJD3 contributes to epigenetic control of INK4a/ARF by oncogenic RAS. Genes Dev.

[156] Agger K (2007). UTX and JMJD3 are histone H3K27 demethylases involved in HOX gene regulation and development. Nature.

[157] Agger K (2009). The H3K27me3 demethylase JMJD3 contributes to the activation of the INK4A–ARF locus in response to oncogene-and stress-induced senescence. Genes Dev.

[158] Hsia DA (2010). KDM8, a H3K36me2 histone demethylase that acts in the cyclin A1 coding region to regulate cancer cell proliferation. Proc Natl Acad Sci.

[159] Ishimura A (2012). Jmjd5, an H3K36me2 histone demethylase, modulates embryonic cell proliferation through the regulation of Cdkn1a expression. Development.

[160] Suzuki T (2006). Tumor suppressor gene identification using retroviral insertional mutagenesis in Blm-deficient mice. EMBO J.

[161] Xu D (2010). Plk3 functions as an essential component of the hypoxia regulatory pathway by direct phosphorylation of HIF-1alpha. J Biol Chem.

[162] Mylonis I (2006). Identification of MAPK phosphorylation sites and their role in the localization and activity of hypoxia-inducible factor-1alpha. J Biol Chem.

[163] Cam H (2010). mTORC1 signaling under hypoxic conditions is controlled by ATM-dependent phosphorylation of HIF-1alpha. Mol Cell.

[164] Karapetsas A (2011). Biochemical and molecular analysis of the interaction between ERK2 MAP kinase and hypoxia inducible factor-1alpha. Int J Biochem Cell Biol.

